# The effects of a Journey of the Brave Counseling Program on anxiety, well-being, and life adjustment in Taiwanese children

**DOI:** 10.1186/s12888-023-05134-8

**Published:** 2023-09-06

**Authors:** Hsiu-Lan Shelley Tien, Yi-Ning Su, Aizi Zhang, Isana Kaichi, Takako Koshiba, Sho Okawa, Yuko Urao, Eiji Shimizu

**Affiliations:** 1 Department of Educational Psychology and Counseling, National Taiwan Normal University, 162 Hoping East Road, Section 1, Taipei, 10610 Taiwan; 2 Research Center for Child Mental Development, Chiba University, 1-8-1, Inohana, Chuo ku, Chiba-shi, Chiba 260-8670 Japan; 3 United Graduate School of Child Development, Osaka University, 2-2, Yamadaoka, Suita, Osaka 565-0871 Japan; 4 Department of Cognitive Behavioral Physiology, Chiba University, 1-8-1, Inohana, Chuo ku, Chiba-shi, Chiba 260-8670 Japan; 5 Department of Experimental Psychology , University of Oxford, Anna Watts Building, Radcliffe Observatory Quarter, Woodstock Road, Oxford, OX2 6GG 272-8533 UK; 6 Japan Society for the Promotion of Science, 5-3-1, Koujimachi, Chiyoda ku, Tokyo, 102-0083 Japan

**Keywords:** Anxiety, Journey of the brave, Life adjustment, SCAS, Well-being

## Abstract

The purposes of the current study are two-fold. Study 1 aimed to examine the psychometric properties of the Spence Children’s Anxiety Scale (SCAS) in a Taiwanese sample. Study 2 aimed to explore the immediate and follow-up effects of Journey of the Brave Counseling Program (JBCP) on children’s’ anxiety, well-being, and life adjustment. A review and suggestions were provided for future research and practitioners in educational and counseling fields as reference. In Study 1, the pilot study included 150 to 200 children between ages 11 and 12 in Taoyuan City. In Study 2, we conducted a pretest-posttest nonequivalent groups quasi-experimental design. The participants in this stage were 16 children in an elementary school in Taoyuan City, between ages 11 and 12. After obtaining consent forms from the participants’ guardians, we randomly assigned these participants to an experimental group (*N* = 8) and a control group (*N* = 8). The experimental group received a 40-minute JBCP session weekly for ten weeks. The control group received a 40-minute career exploration small group counseling weekly for ten weeks. We administered the SCAS, Psychological Well-Being Scale, and School Life Adjustment Scale in the pretest, posttest, and follow-up test to measure change of anxiety, well-being, and life adjustment of the participants. In addition, the current study implemented some qualitative data, such as group progress notes, group member feedback questionnaires, and semi-structured interviews with participants’ homeroom teachers as supplementary data to clarify the effects of the JBCP. In Study 1, we found that the SCAS had a good validity and reliability for Taiwanese children. The results of Study 2 indicated that the JBCP had immediate and follow-up effects on the separation anxiety in the experimental group. With the pretest impact eliminated, the immediate and follow-up effects on overall anxiety in the experimental group were better than those on the control group. However, even though the immediate and follow-up effects of the JBCP on the experimental group were better than the control group but were not significant. Besides, the group member feedback questionnaires and participants’ homeroom teachers all indicated that the experimental group participants had positive attitude toward the JBCP, and they also positively improved their emotions and interpersonal relationships with others.

## Background

As social changes, people’s life nowadays is getting more and more compact with increasing stress led to their daily lives. In particular, the raging of the COVID-19 epidemic has impacted all industries and people’s ordinary lifestyles, seriously affected economic and living conditions of numerous families. Under such critical background, many children are lack in a safe growing environment for their parents may need to make their ends meet in their daily life. For children and adolescents who undertake drastic change in all aspects during growth, a stable growing environment and significant other’s support are greatly needed. Without these, all kinds of inner development and life adjustment in children will be deeply influenced. In a long run, the children may suffer various obstacles and difficulties in life mentally and physically. Many statistics data report emotional and life maladaptation are found in some children and adolescents. A study on adolescents’ life conducted by the Accounting Office of the Executive Yuan in 2016 indicated that 55.30% of 15- to 29-year-old adolescents and young adolescents encountered problems in life (Directorate General of Budget, Accounting and Statistics, Executive Yuan, 2016) [8]. According to a survey on the health right of the Taiwanese children and youths (Taiwan Fund for Children and Families, 2012) [39], about one-third of the participants suffer from emotional distress and tend to have suicidal ideation.

In the elementary school where one of the authors works, an increasing number of students are found to suffer from emotional distress, which is reflected on these children’ schoolwork, interpersonal relationship with their peers, and the school activities they participate in—all of which cause a great deal of problems in life adjustment. We notice that the students who encounter problems of emotional distress are not aware of their own emotional perception. If there was no timely intervention and care, these suffering children would not know how to cope with their anxiety and feelings. This vicious cycle in these children may repeat, accumulating negative experiences, and this may turn into serious mental issues. Therefore, problems of anxiety and life adjustment in children and adolescents need highly valuing. If someone provides an appropriate counseling intervention, take precautionary measures for the children in need, or identify the problems in an early stage and actively offer intervention treatment, anxiety or adjustment issues may be more effectively prevented or relieved than they are in one’s adolescence or adulthood.

## Anxiety and cognitive behavior

Anxiety is an important sense during the development of human growth. It allows people to be alerted when facing restless and dangerous situations and hence improves one’s life adjustment ability. However, once accumulated anxiety occurs constantly or lasts long, people may suffer serious mental disorder, resulting in mental health risks. Therefore, anxiety level is one key element that mental health workers care with (Lau et al., 2010) [20], and it is an important index to evaluate individuals’ mental health. Seligman (2011, 2019) [31] [32] initiated the promotion of positive psychology, which is a concept focusing on viewing issues in a positive perspective. Individuals may remain a more positively and actively flourishing status in their personal or social life to fulfill the goal of pursuing an authentic happiness and beautiful life by improving their well-being and positive feelings to make them energetic and enthusiastic toward their life. It focuses on individuals’ positive aspects and strengths as a path to wellbeing. In positive psychology, well-being is a key concept that many researchers have been focusing on when exploring mental health issues (Yu et al., 2017) [50], which shows well-being is a key index. Invotive ideas about positive psychology include hope, resilience, flow, and strength theories are all great components applied in counseling (Sutton, 2023) [38].

The discussions above show that both positive emotions and negative emotions are set as criteria for people to assess individuals’ mental health. There is a close relationship between individuals’ mental health and adjustment into life. Studies in the past reported that different emotions emerge from one’s experience, thoughts, and perceived situational stimuli. Individuals may adjust the process of emotion arousing to increase or decrease positive or negative emotions (Gross, 2007) [11]. Therefore, individuals’ awareness and cognition of situations and their adaptability of emotions may influence the strength of individuals’ positive and negative emotions and further make difference on one’s mental health and life adjustment.

### Development of a cognitive behavioral therapy program for children

Cognitive Behavioral Therapy (CBT) is commonly used to treat anxiety (Barlow, 2002; Kendall et al. 2004) [2] [17]. Many studies conducted in the world confirm the effectiveness of CBT for anxiety (Bennett et al., 2013; Ewing et al., 2015; Öst & Ollendickc, 2017) [5] [10] [29], and they report that CBT treatments are of significant counseling effects, effectively reducing symptoms of anxiety in individual. However, Wu (2020) [45] found that only 18 empirical studies on CBT for anxiety were conducted in Taiwan, within which five were for children. There are insufficient studies about CBT for group counseling, which indicated a lack of empirical studies in Taiwan. Therefore, we decided to conduct a study on the effectiveness of CBT for group counseling. Chiba University in Japan constructed a cognitive behavioral program called Journey of the Brave, aiming to deal with emotional disturbance in children and adolescents. The program was reported to be effective in reducing anxiety and depression in Japanese children and adolescents and it is not implemented in a Taiwanese context. Considering cultural and regional characteristics, the current study aimed to explore the effectiveness of Journey of the Brave program in elementary school 5th and 6th graders between 11 and 12 years old. Besides, with the professor at Chiba University and under the evaluation of the authors, we adopted the Spence Children’s Anxiety Scale (SCAS; Spence, 1998) [36] as instrument to assess anxiety symptoms in children, hoping to confirm the applicability of the SCAS in Taiwan.

### Purpose of the study

The current study aimed to (1) examine the applicability of the SCAS (Spence, 1998) [36] in Taiwanese children; (2) examine the effectiveness of the Journey of the Brave counseling program (JBCP) in reducing anxiety symptoms in children as well as promoting their well-being and life adjustment. Therefore we address four research questions.

1. What are psychometric properties of the SCAS in a Taiwanese sample?
2. Does the JBCP have immediate and follow-up effects on anxiety symptoms in elementary school students?
3. Does the JBCP have immediate and follow-up effects on the well-being of elementary school students?
4. Does the JBCP have immediate and follow-up effects on the life adjustment of elementary school students?

## Literature Review

### Cognitive behavioral therapy and anxiety symptoms

Anxiety symptoms refer to two emotional reactions: anxiety and fear. The former is a reaction when one is overly concerned about a future problem, while the latter is an emotional reaction used to confront immediate danger. These two feelings stimulate sympathetic nervous system, resulting in some mental behavior reaction, such as restlessness, muscle tension, shortness of breath, and eagerness to run away (Kring et al., 2014) [18]. The heterogeneity in children’s anxiety is high, and many factors may affect children’s anxiety disorders. These factors’ forming processes are similar, and the most discussed ones include negative affectivity, perception of control, specific life experiences, and anxious thinking (Yen, 2010a) [47]. Negative affectivity is a temperament risk factor for anxiety disorder. Children with a high level of negative emotion traits are inclined to be sensitive to negative events and threatening objects and messages, so they tend to easily feel nervous, worried, scared, or sad. Hsieh et al. (2018) [13] confirmed that the higher level of children’s negative emotions and extraversion are, the greater tendency they have to feel anxious and depressed. As for perception of control, children tend to have negative reaction toward uncontrollable things or threatening events. The perception they have when experiencing these uncontrollable events may limit children’s opportunities to explore the world, confront challenges, and ask for help at right time, which may cause the children have a great anxiety feeling. To overcome anxiety feelings, children must develop a sense of control with which they may feel they are capable of getting rid of or appropriately confronting awful things (Kendall & Treadwell, 1996) [16]. Specific life experiences occur in different children with different negative emotion traits, and children develop different anxiety symptoms. For example, once a child with a high level of negative emotion traits got bitten by a dog, this child may show a specific phobia toward dogs. Children with anxious thinking tend to mention negative information. Such traits may cause deviation in attention, interpretation, and self-talk, which may lead to a vicious circle of negative thoughts and anxious feelings in children.

Negative affectivity in early childhood may influence children’s perception toward the environment and further bring negative recognition tendency toward early experience such as an experience of a sense of control and towards specific life experience, manifesting negative cognitive disposition in children (Barlow, 2002) [2]. Overall, all, anxiety feelings in children are greatly under the influence of the events they experience and their cognition.

### Cognitive behavioral therapy for children’s anxiety symptoms

Cognitive Behavioral Therapy (CBT) is a treatment method developed from the treatment for patients with depression. Beck et al. (1979) [4] believe that individuals’ depression is usually influenced by the intermediate beliefs like attitudes or schemes developed in past experiences, resulting in automatic thoughts that disturb individuals. Therefore, the development pattern of a negative cognitive process is the same as the cognitive process of children’s anxious feeling, indicating that CBT may effectively help children be aware of their automatic thoughts, beliefs, and emotions and identify cognitive bias, and reduce psychological discomfort by changing children’s cognitive process (Kaplan et al., 1995) [15].

Imperial studies in Taiwan also report that CBT has therapeutic effectiveness in treating children’s anxiety disorders. Zhang (2006) [52] points that during the process of CBT, children and adolescents may learn to control anxiety feelings and their perception toward events under the guidance on dealing with anxious events and providing opportunities to practice the skills. During the CBT, counselors may take notes to explore children’s life experience and way of thinking. With a structured therapy note, children may clearly record their life experience. Along with Socratic questioning, counselors may help children beware of their emotions and feelings, inspect the proofs of automatic thoughts, and then find out their cognitive bias (Wu, 2020) [45]. In addition, implementing CBT for children into mental health education is effective in improving anxiety symptoms (Barrett & Turner, 2001; Yen et al., 2012) [3] [49]. Wu (2020) [45] and Tsai (2012) [40] also indicate that adopting CBT for children in a specific psychological health education course or the use of picture books or tory books have significant effects on improving children’s cognitive development.

### Cognitive behavioral therapy for well-being, and Life Adjustment

#### Well-being

Primarily proposed by Andrews and Withey (1976) [1], the concept of well-being is an individual’s subjective experience including three components, life satisfaction, positive feelings, and negative feelings, which are combined with two dimensions, affection and cognition. Contemporary psychology studies related to well-being include psychological well-being and subjective well-being. Subjective well-being researchers believe that subjective well-being is equivalent to happiness, while psychological well-being researchers refute the excessive attention paid to emotions by subjective well-being. Psychological well-being researchers suppose that well-being is the result of an individual’s comprehensive evaluation of their life-quality on a self-determined standards and produce a relatively stable cognition and emotion experience. Therefore Ryff (1989) [30] developed a psychological well-being model, which include six facets as follows: (1) Autonomy, the ability to overcome social pressure and think and act independently; make judgement and decisions based on the standard determined by oneself. (2) Environment management, the ability to manage external complex environments; efficiently utilize limited environmental resources to select and create conditions that can enhance personal values. (3) Personal growth, the ability to try new things and fulfill one’s potential, making one to be in the process of continuous growth and development. (4) Positive interpersonal relationship, the ability to maintain a harmonious, sincere interpersonal relationship. (5) Purpose of life, the ability to have a goal and direction for life and have beliefs in life. (6) Self-acceptance, being able to accept one’s own strengths and weaknesses as well as behold a positive attitude toward oneself. Therefore, a stable cognitive emotion experience is developed to enhance one’s psychological well-being, individuals may like themselves more, establish a warm and trustworthy relationship with others, actively manage environment resources, and have a specific goal to obtain for self-growth and realization. In recent years, Güleç Keskin and Gülirmak (2022) [12] also found that the effect of psychoeducation of the positive psychotherapy based balance model could be applied to students on spirituality level of subjective well being.

#### Life Adaptation

Adaptation is derived from Darwin’s (1895) [6] theory of evolution in which the mechanisms, natural selection, the superior eventually defeating the inferior, and survival of the fittest, are mentioned. All creatures need to adequately adapt themselves to fit their living environment. Piaget believes that adaptive individuals may continuously integrate new and old experience through assimilation and accommodation and by merging and adjusting their inherent cognitive to achieve a balance with the external environment (Zhang, 1996) [51]. Therefore, life adaptation is not only a purpose but also a process. Within the process, individuals interact with the environment to maintain a harmonious status and life adaptation is the ability cultivated to cope with environmental changes (Kung, 2013) [19].

#### CBT for well-being and Life Adaptation

Welling being and life adaptation has always been the focus of counseling practice. We believe that well-being is closely related to the individuals’ cognition and interpretation of life experiences. Lin (2011) [24] found that personality traits can influence the individual’s perception and memory about certain events. When we think about something in a positive way, we may feel of happiness and would be able to adjust better in facing challenges and frustrations.

Cognitive psychologists argue that individuals are not a passive system to receive messages but an active one to spontaneously interpret external messages (Wittrock, 1990) [44]. The impacts of all incidents occur to individuals need to pass through a system of self-awareness and evaluation to affect behavioral outcomes and emotional feelings. Smith and Lazarus (1990) [35] proposed a model of the cognitive-motivational-emotive system, emphasizing that emotions produced during the interaction between individuals and the environment. This model suggests that individuals’ internal factors and situational factors are the two variables that affect the emotional process. Therefore, when interacting with the environment, individuals are inclined to establish appraisal and coping abilities. If an individual interacts with the environment, the negative emotional cognition generated by the negative cognitive model will affect the individual’s perception of the environment as well as limit the individual’s positive emotional experience toward the environment and cause unseen psychological problems for future (Xie et al., 2018) [45]. On the contrary, if an individual interacts with the environment, the individual’s stable internal cognitive model may affect the appraisal and coping abilities toward the environment and then further affect the emotional cognitive and the interpretation of the environment, producing a more positive emotional cognition and emotions. Therefore, CBT is used to adjust an individual’s internal automatic negative cognition model and establish a more stable internal emotional cognition through cognitive awareness and reconstruction. In this way, individuals may improve their connection and evaluation of the positive perception of the situation, and create a harmonious interaction with the environment, fostering life adaptability, and achieving a great well-being and successful adaptation to life.

### Cognitive behavioral therapy program

Group and individual intervention treatments are common methods adopted in psychotherapy or guidance and counseling services, but the differences lay not only in the number of clients but also the effects during the intervention process. Yalom (1995) [46] classified the effect of group counseling into 12 categories, among which group counseling has the most distinctive counseling effects in its universality, interpersonal learning, behavioral imitation, socialization skills developing, cohesiveness. Compared with individual intervention treatment, group counseling offer a relatively secure environment to group members. Group counseling clients may obtain support through interaction with others in a relatively secure environment. While increasing their sense of comorbidity, group counseling clients may also reinforce the motivation and willingness to make cognitive change and practice in a scenario simulation session (Yalom, 1995) [46]. Studies in the past show that CBT group therapy has significant effects on anxiety disorders, such as generalized anxiety disorder, social anxiety disorder, panic disorder and other anxiety- related disorders (Shortt et al., 2001) [34]. This indicates that CBT group counseling also has therapeutic effects on clients with emotional distress, so there is a need to develop a CBT ground counseling program.

## Study 1: validation of the Spence children’s anxiety scale -Taiwan version

The Spence Children’s Anxiety Scale (SCAS) was established by Spence (1998) [36] to promote relevant research and evaluate counseling practice. It was designed to evaluate the severity of anxiety disorders in children. Although the SCAS is not the only instrument to measure anxiety disorders, it is used to identify the children at risk of potential anxiety disorders, serving as an instrument to prevent anxiety disorders or monitor the intervention effectiveness in treating anxiety disorders. In clinical practice, the SCAS has been widely adopted in the assessment and treatment of anxiety disorders.

The development of the SCAS used children in the community as a sample, based on Diagnostic and Statistical Manual of Mental Disorders, 5th edition (DSM-V) to assess six anxiety symptoms in children, including panic/agoraphobia, separation anxiety, physical injury fears, social phobia, obsessive compulsive disorder, and generalized anxiety. The SCAS consists of 45 items, scored on a 4-point Likert Scale (Never = 0; Sometimes = 1; Most of Time = 2; and Always = 3). The participants will respond to the questions according to their own experience. The scores for the subscales and the full scale are calculated respectively. The higher the scores are, the higher level of the anxiety symptoms the participants have.

### Validation of the Spence children’s anxiety scale

The SCAS has been widely adopted and validated in many countries and regions in the world under various cultural contexts, such as in Australia (Spence, 1998; Spence et al., 2003) [36] [37], Netherland (Muris et al., 2002) [27], the USA (Whiteside & Brown, 2008) [43], Japan (Ishikawa et al., 2009) [14], Hong Kong (Li et al., 2011) [23], China (Zhao et al., 2012) [53]. The SCAS reported good reliability and validity not only in the study of Western culture but also in the study of Eastern culture. Ishikawa et al. (2009) [14], Spence et al. (2003) [37], and Spence (1998) [36] all confirmed that the SCAS has stable internal consistency and good test-retest reliability. The convergent and discriminative validity of the SCAS also have been confirmed. The significant correlation between the SCAS and other anxiety disorder scales or measurements, such as Revised Children’ s Manifest Anxiety Scale, RCMAS, and The Screen for Child Anxiety Related Emotional Disorders, SCARED, is much greater than the measurements for depression in children (Muris et al., 2002) [27], indicating good discrimination of the SCAS. Inconclusion, we believe that SCAS serves as a reliable and valid tool for assessing anxiety symptoms in children.

The SCAS has been translated into many languages, including Hong Kong-published Chinese-translated version of the SCAS (the SCAS-HK; Wang & Deng, 2004) [41]. However, the assessments and results may differ due to different languages and cultures. For example, the SCAS is reported that there is a difference between the sample in Japan and that in Germany (Essau et al., 2004) [9]. Besides, the psychometric properties of the SCAS are different in the sample of Australia (Spence, 1998) [36] and Germany (Essau et al., 2004) [9]. Therefore, even though Taiwan and Hong Kong share similar language contexts that are under the influence of Chinese culture, historical and cultural changes in Taiwan and Hong Kong may have developed their own unique culture contexts. We needed to examine whether the SCAS-HK, is effective in assess anxiety symptoms in Taiwanese children.

## Method

### Participants

Study 1 aimed to validate the subscales and full scale of the SCAS, using the Multidimensional Anxiety Scale for Children-Taiwanese Version (MASC-TV), as criteria to verify the criterion-related validity of the SCAS for anxiety symptoms in Taiwanese children. The participants were 188 11-years and 12-years old elementary school students in Taoyuan City, Taiwan, and the test was conducted on the basis of a school class size in the academic year of 2021.

### Measurements

#### The SCAS-HK

The SCAS-HK (Wang & Deng, 2004) [41] was used as the instrument in Study 1. This version was analyzed and validated in Hong Kong (Li et al., 2011) [23] and China (Zhao et al., 2012) [53]. Both studies indicated that the SCAS, Hong Kong Version, has high internal consistency. Zhao et al. (2012) [53] reported that the SCAS has satisfactory test-retest reliability and the subscales of the SCAS have moderate to good internal consistency reliability and test-retest reliability, which showed that the SCAS has good reliability. Besides, factor analysis in both studies showed that the six factors of the SCAS, Hong Kong Version, produced reasonable results in the sample with good factor structure and good construct validity. In addition, Li et al. (2011) [23] reported the total scores of the SCAS, Hong Kong Version, was significantly correlated with the anxiety subscale of the Negative Affect Self Statement Questionnaire (NASSQ). Zhao et al. (2012) [53] also reported that the subscale and full scale of the SCAS, were highly correlated with the Screen for Child Anxiety Related Emotional Disorders (SCARED). Both studies indicated that the SCAS—has good convergent validity.

#### MASC-TV

The MASC-TV (Yen, 2010b) [48] effectively assesses anxiety-related symptoms in children and adolescents. The MASC-TV is a 39-item scale consists of four major constructs, three of which can be divided into two sub-constructs. The major constructs include physical symptoms (tense/restless and somatic/autonomic), harm avoidance (anxious coping and perfectionism), social anxiety (humiliation/rejection and public performance fears), and separation anxiety.

The MASC-TV is scored on a four-point scale; the higher the scores, the higher the level of anxiety symptoms is in participants. The internal consistency coefficient of each construct and index were between 0.62 and 0.85; the test-retest reliability of each construct was between 0.66 and 0.89, which indicate a good reliability. Besides, factor analysis for the MASC-TV showed that the four-factor structure best fit the data and have good construct validity. In addition, the scores of the children with anxiety symptoms are significantly higher than those of the children without anxiety symptoms, which showed good discriminant validity.

## Results

### Background differences on SCAS subscale

In study 1, we conducted ANOVA to examine the differences of gender and age for the SCAS, using gender and age as independent variables and the scores for the SCAS as a dependent variable. The analysis reported that score for gender, (F[1,184] = 0.60, *p* = .01), *p* < .05, is significant, which indicates that the scores of different genders for the SCAS have significant differences. The scores for age, (F[1,184] = 6.41, *p* = .44), *p* > .05, is not significant, which indicates that there is no significant difference in the scores for the SCAS-HK of children at different ages. Besides, the scores of gender and age, (F[1,184] = 0.12, *p* = .73), *p* > .05, are not significant, which means that the scores for the SCAS of children at different ages are not significant and there is no significant interaction between genders and ages. In conclusion, the scores for the SCAS of girls are significantly higher than those of boys, which shows that girls tend to have a higher level of anxious disorders than boys. However, the scores for the SCAS of different ages are not significant, and the scores of different genders do not make difference with different ages.

Using gender as an independent variable and scores for the SCAS subscales as dependent variables, the results of ANOVA were shown in Table 1. The gender differences for both social phobia and generalized anxiety are significant statistically (F[1,186] = 8.33, *p* = .00 for social phobia and F[1,186] = 5.39, *p* = .02 for generalized anxiety). Both *p*-values are less than 0.05, being statistically significant. The result shows that there is a significant difference in different gender for the scores for social phobia and generalized anxiety, which shows that girls scored significantly higher than boys in social phobia and generalized anxiety.

**Table 1:** ANOVA test on SCAS using gender as an independent variable

Item	Boys		Girls			F	P
	M	SD	M	SD			
Panic/Agoraphobia	2.83	3.33	3.62		3.89	2.23	0.14
Separation Anxiety	5.23	3.47	6.14		3.98	2.74	0.10
Physical Injury Fears	5.26	3.23	6.05		3.03	3.01	0.09
Social Phobia	5.89	3.66	7.52		4.06	8.33	0.00^**^
Obsessive Compulsive Disorder	5.61	3.47	6.34		3.97	1.82	0.18
Generalized Anxiety	4.50	3.13	5.65		3.64	5.39	0.02^*^
Full Scale	29.32	14.82	35.32		17.47	6.42	0.01^*^

Nunnally (1978) [28] suggested that an instrument is considered high reliability if Cronbach’s alpha coefficient above 0.7. However, if the Cronbach’s alpha coefficient is lower than 0.35, the instrument is considered as low reliability should be rejected. The analysis in Study 1 reported that the Cronbach’s alpha values for the Panic/Agoraphobia, Separation Anxiety, and Social Phobia subscales are 0.77, 0.70, and 0.73 respectively, indicating a high reliability. Besides, the Cronbach’s alpha values for the Physical Injury Fears, Obsessive Compulsive Disorder, and Generalized Anxiety are 0.49, 0.68, and 0.52 respectively, indicating that they have acceptable internal consistency. The Cronbach’s alpha value for the SCAS is 0.88, which indicate that the scale has good internal consistency.

### Criterion-related validity analysis

The results of the correlation coefficient analysis between the SCAS and MASC-TV are shown in Table 2. The correlation efficient between the SCAS and MASC-TV is 0.81, and the *p* value is less than 0.01, reaching statistical significance, which indicates that there is a significant correlation between the SCAS and MASC-TV. The correlation coefficient of the Physical Injury Fears subscale of the SCAS and the Harm Avoidance subscale of the MASC-TV was not significant, which indicate that there is a difference needed to be discussed in a Taiwanese sample. The correlation between the Physical Injury Fears subscale of the SCAS and the Harm Avoidance subscale of the MASC-TV in the study of Muris et al. (2002) [27] was also relatively low, compared with other dimensions. However, the correlation coefficient between the subscales of the SCAS and those of the MASC-TV is statistically significant, which indicates that there is a positive correlation between the two scales in most dimensional measurement., using the MASC-TV and the subscales of the MASC-TV as criterion, the SCAS and the MASC-TV are highly correlated. This shows that the SCAS and the SCAS subscales were demonstrated to have good criterion-related validity.

**Table 2:** Correlation matrix of the Spence Children’s Anxiety Scale (SCAS) and Multidimensional Anxiety Scale for Children-Taiwan Version (MASC-TW)

	SCAS						
MASC-TV	Panic/ Agoraphobia	Separation Anxiety	Physical Injury Fears	Social Phobia	Obsessive Compulsive Disorder	Generalized Anxiety	Full Scale
Physical Symptoms	0.67^**^	0.44^**^	0.33^**^	0.45^**^	0.42^**^	0.61^**^	0.64^**^
Tense/Restless	0.65^**^	0.46^**^	0.39^**^	0.48^**^	0.44^**^	0.66^**^	0.68^**^
Somatic/Autonomic	0.57^**^	0.34^**^	0.20^**^	0.34^**^	0.34^**^	0.44^**^	0.49^**^
Harm Avoidance	0.23^**^	0.24^**^	0.08	0.34^**^	0.50^**^	0.28^**^	0.37^**^
Perfectionism	0.20^**^	0.18^*^	0.06	0.36^**^	0.42^**^	0.23^**^	0.33^**^
Anxious Coping	0.20^**^	0.24^**^	0.08	0.24^**^	0.47^**^	0.26^**^	0.32^**^
Social Anxiety	0.49^**^	0.50^**^	0.44^**^	0.79^**^	0.51^**^	0.58^**^	0.74^**^
Humiliation/Rejection	0.45^**^	0.44^**^	0.37^**^	0.76^**^	0.52^**^	0.52^**^	0.68^**^
Public Performance Fears	0.44^**^	0.49^**^	0.45^**^	0.67^**^	0.38^**^	0.55^**^	0.66^**^
Separation Anxiety	0.52^**^	0.71^**^	0.49^**^	0.47^**^	0.38^**^	0.55^**^	0.68^**^
Full Scale	0.63^**^	0.62^**^	0.45^**^	0.70^**^	0.60^**^	0.67^**^	0.81^**^

^*^p < .05, ^**^p < .01, N = 188

## Study 2: the Effects of the JBCP on children’s anxiety, Well-Being, and Life Adjustment

### Method

In Study 2, we conducted a pretest-posttest nonequivalent quasi-experimental design. We recruited participants aged between 11 and 12 in an elementary school in Taoyuan, Taiwan. After obtaining the consents of the participants and their parents, we randomly assigned the participants into the experimental group and the control group. The experimental group and the control group received different intervention treatments. The former received a 40-minute Journey of the Brave group counseling session weekly for ten weeks. The latter received a 40-minute career exploration session weekly for ten weeks. Prior and posterior to, and one month after the group counseling treatment, we administered the SCAS-TW, Psychological Well-being Scale, and School Life Adjustment Scale to the participants in the pretest, posttest, and follow-up tests to examine the immediate and follow-up effects. It took about 20–25 min in class to finish the survey. In addition, we also implemented group progress notes, group member feedback questionnaires, and interview with the participants’ homeroom teachers to obtain supplementary data for the study.

### Participants

The participants in Study 2 were 16 children aged between 11 and 12. We also invited the participants’ homeroom teacher as participants. For the 16 children, each of them were assigned a number from 1 to 16. We then drew 8 numbers randomly and the eight students were assigned to experimental group. The rest 8 participants were then assigned to control group. The experimental group (*N =* 8) included three boys and five girls; while the control group (*N =* 8) include two boys and six girls. After the treatment, we conducted semi-structured interview with 5 homeroom teachers who consented to be interviewed. Those homeroom teachers observed and understood the participants’ daily behaviors, emotional feelings, and life adjustment at school.

### Instruments

#### The Journey of the Brave Counseling Program

The study design of Study 2 was based on a CBT based programme “Journey of the Brave” developed by the Research Center for Child Mental Development of Japan Chiba University. After reviewing relevant CBT studies and the development status of elementary school 5th and 6th grade student between the ages of 11 and 12, combing authors’ group counseling experience, we established a Journey of the Brave Counseling Program (JBCP) for children in Taiwan. The JBCP is based on CBT theories, aiming to guide children to understand and confront anxious feelings (show as in Table 3). During the process of learning what anxious feelings and emotions are, children may explore the relation among thoughts, emotions, and behaviors to understand the emotional process and context of being restless and anxious. Through discussions on restless and anxious feelings and situations, children may enhance the awareness of their irrational beliefs and transfer their thoughts and emotions as well as learn to face and adjust their anxious feelings. In addition to some cognitive thinking practice, some practical methods are added into the JBCP, and children practice how to break anxious feelings into specific fragments by systematically checking sensitivity, along with some relaxation technique and interpersonal relation skill developing exercises, to help children gradually face anxious feelings.

**Table 3:** The Purpose and Goal of the Journey of the Brave Counseling Program

Unit	Title	Goal	Content
1	Preparation	1. Understanding the nature and the purpose of the program 2. Assisting group members in knowing one another 3. Setting up the rule for the group together	1. Understanding the Journey of the Brave 2. Meeting and greeting 3. Our appointment
2	Start-off	1. Understanding emotions 2. Understanding the way to express emotions 3. Knowing miracle scores	1. Emotional words competition 2. Changeable emotions 3. Miracle scores 4. Time to share
3	Plan	1. Knowing anxious feelings 2. Exploring the cause make oneself uneasy and the degree of the uneasiness 3. Setting the goal of the Journey of the Brave	1. Anxious feeling 2. The goal for the Journey of the Brave 3. Time to share
4	Basic Skill-Muscle Relaxation	1. Understanding anxious feelings and physical reaction 2. Finding out what to do when knowing physical reaction 3. Practicing the way of relaxation	1. Top challenge 2. Physical reaction 3. Relaxation practice 4. Time to share
5	Applied Skill-A Ladder for the Brave	1. Understanding the trap that anxiety sets up 2. Understanding that facing the anxiety is the best solution 3. Building up the brave’s ladder	1. The tips for anxious feelings 2. Building up a ladder for the Brave 3. Time to share
6	Developed Skill- the Brave’s Triangle	1. Understanding the relation among thoughts, emotions, and behaviors 2. Creating a triangle for the brave	1. A quiz game 2. Knowing the brave’s triangle 3. Creating a triangle for the brave 4. Time to share
7	The Naughty Elf that Confuses the Brave	1. Understanding the thoughts in mind when feeling anxious and the knowing the types of anxiety 2. Understanding the impact of the anxiety 3. Understanding how to resolve the anxious feeling	1. Naughty Elf’s words 2. A magic trick-running round in circles 3. The secret to break the magic trick 4. Time to share
8	An Ultimate Weapon of the Brave-How the Brave Thinks	1. Knowing the brave’s way of thinking 2. Practicing knowing the brave’s way of thinking 3. Practicing thinking of the reason whey one feels anxious 4. Practicing reviewing how one thinks	1. Knowing the brave’s way of thinking 2. Examining the Naughty Elf’s words 3. The brave’s way of thinking 4. Time to share
9	Secret Skills of the Brave-the Way the Brave Talks	1. Knowing the anxious situation within interpersonal relation 2. Practicing interpersonal communication skills	1. Scenarios story 2. How the brave talks 3. Other skills practice 4. Time to share
10	A Test at the End of the Journey of the Brave	1. Reviewing the group counseling process 2. Sharing feedbacks with group members	1. The Journey of the Brave counseling session reviews 2. Feedback time 3. Closing time

#### SCAS-TW

We used the SCAS-TW developed in Study 1.

#### Psychological well-being scale (PWS; Lee, 2010)[22

Study 2 used the PWS to examine children’s psychological well-being. The PWS consists of six constructs, namely self-acceptance, autonomy, environment control, self growth, positive interpersonal relationship, and purpose of life. The 36-item PWS is rated on a 4-point Likert’s scale (strongly disagree = 1, disagree = 2, agree = 3, strongly agree = 4), within which are four reverse scoring items. The higher the scores are, the higher degree of psychological well-being. The internal consistency reliability of the PWS is 0.03, and the Cronbach’s alpha coefficient is 0.93. Factor analysis reported that the factor loadings of each item is above 0.70, and the six factors could explain 77.81% of total sum of square of the 36-item scale, indicating good reliability and validity.

#### School life adjustment scale (SLAS; Wang et al., 2017)[42

The SLAS is used to assess children’s adjustment on all aspects situations at school. The SLAS was developed in 2012 by the authors, consists of 3 subscales, the Adjustment on the Teacher-Student Relationship (TSR), Adjustment on the Self-Peer Relationship (SPR), and Adjustment on the Academic Challenge (AC). This is a 12-item scale, rated on a 4-point Likert scale (strongly disagree = 1, disagree = 2, agree = 3, and strongly agree = 4). The AC subscale is reverse-scored. The higher the scores are for the SLAS, the better the children’s school life adjustment is. Confirmatory factor analysis reported that the scale had a stable two-factor structure (Bi-Factor Model; RMSEA = 0.06, SRMR = 0.05, CFI = 0.97). There was a positive moderate correlation between the three subscales of the SLAS and confidence and the ability to solve problems, and a negative correlation with internal and external behaviors, indicating that the scale had good criterion-related validity.

Other instruments applied in Study 2 were group member feedback questionnaires, interview portfolios, and group progress notes. Group member feedback questionnaires were developed by one of the authors, aiming to understand participants’ experience and feedback after they receive group counseling session treatments. The questionnaire includes three sections. The first section asked about participants’ basic information. The second section asked about participants’ feedback about the group activities they have taken part in, rated on a 5-point Likert scale. The third section consists of open questions asking about participants’ reflection on participating in group counseling sessions. Another instrument we used in Study 2 were interview portfolios for semi-structured interviews with the experimental group participants’ homeroom teachers. In the interviews, the homeroom teachers shared what they observed from the participants about their emotions, interpersonal relation, and changes after they received the JBC treatment. The group progress notes were taken after every counseling treatment, in which counseling leaders made records of every counseling session process and the reaction of group participants to evaluate the efficiency of the group counseling and participants’ behaviors and statuses. The counseling leader is a researcher, with a background in psychological counseling and experience in leading group counseling, and is currently working as a tutor in a primary school.

### Data Analysis

In Study 2, we analyzed the data collected from the scores of the experimental and control groups on the pretests, posttests, and follow-up tests for the SCAS-TW, PWS, and SLA. The quantitative data were analyzed via analysis of variance to test the hypotheses. As for the qualitative data, we conducted content analysis to transcribe, code, and analyze the data collected from group feedback questionnaires, semi-structured interviews, and group process notes. The data would be used as supplementary data to evaluate the effectiveness of the group counseling.

## Results

### The immediate effects of the JBCP on anxiety, well-being, and life adjustment in Taiwanese children

#### Anxiety

We conducted the test of homogeneity of within-group regression coefficient to eliminate the existence difference between the experimental and control groups. This was to reduce the impact of the differences on the pretest, and then we carried out analysis of covariance to compare the differences between the experimental and control groups in the pretests and posttests for and of the SCAS and subscales and reduced the impact of the pretests. The test score of homogeneity of within-group regression coefficient of the experimental and control groups on the posttest for the SCAS scale and subscales, (F [1, 12] = 3.19, 0.36, 0.02, 0.14, 0.00, 0.12, 0.00, *p* > .05), did not reach a significant level, indicating that the slopes in the posttest of the experimental and control groups for the SCAS scale and subscales were the same. The result was consistent with the assumption of homogeneity of regression coefficient, and then we preceded to conduct analysis of covariance. The analysis of covariance reported that the scores of the experimental and control groups in the posttest on the SCAS scale (F [1, 13] = 1.38, *p* = .26, *p* > .05) did not reach a significant level. However, the scores of the experimental group on the posttest for the Separation Anxiety subscale of the SCAS, (F [1, 13] = 7.06, *p* = .02, *p* < .05), were significantly lower than the control group. This indicated that after the group counseling treatment, the anxiety level of the experimental group participants was significantly than the control group.

After eliminating the pretest effect, the average scores of the experimental group on the posttest after adjusted for the SCAS scale and subscales were lower than those of the control group (As shown in Table 4). This indicated that even though the posttest scores of the experimental and control groups was not significant, with the experimental treatment, the overall anxiety level of the experimental group were lower than that of the control group, which confirmed that the JBCP had immediate effect on anxiety in children.

**Table 4:** Means and Standard Deviation of the Pretest and Adjusted Posttest of the Experimental Group and the Control Group for the Spence Children’s Anxiety Scale

Item	Experimental Group (*N* = 8)			Control Group (*N* = 8)		
	Pretest	Posttest	Posttest (Adjusted)	Pretest	Posttest	Posttest (Adjusted)
	M (SD)	M (SD)	M (SE)	M (SD)	M (SD)	M (SE)
Panic/ Agoraphobia	4.75 (4.27)	2.75 (2.71)	4.07 (1.69)	8.63 (4.27)	6.25 (6.88)	4.93 (1.69)
Separation Anxiety	6.25 (4.77)	3.88 (4.85)	3.93 (0.97)	6.38 (4.00)	7.63 (4.34)	7.51 (0.97)
Physical Injury Fears	4.63 (4.47)	2.63 (2.50)	3.41 (1.02)	8.13 (2.70)	5.63 (3.58)	3.84 (1.02)
Social Phobia	7.25 (4.20)	4.75 (3.73)	5.13 (0.86)	8.38 (5.29)	7.75 (4.20)	7.31 (0.86)
Obsessive Compulsive Disorder	5.25 (4.27)	3.13 (3.18)	3.26 (1.41)	8.38 (5.63)	6.00 (4.28)	5.87 (1.41)
Generalized Anxiety	4.25 (3.01)	3.75 (3.28)	4.70 (0.76)	6.25 (3.01)	6.38 (3.70)	5.42 (0.76)
Full Scale	32.38 (20.33)	20.88 (18.92)	25.92 (5.02)	46.13 (13.81)	39.63 (17.63)	34.58 (5.02)

#### Well-being

The test of homogeneity of within-group regression coefficient reported that the scores of the experimental and control groups in the posttest on the PWBS scale and subscales, (F [1, 12] = 0.11, 0.97, 0.87, 0.40, 0.57, 0.35, 0.07, *p* > .05), did not reach a significant level, indicating that the slopes in the posttest of the experimental and control groups for the PWBS scale and subscales were the same. The result was consistent with the assumption of homogeneity of regression coefficient, and then we preceded to conduct analysis of covariance to compare the difference between the experimental and control groups in the pretest and posttest on the PWBS scale and subscales. However, the analysis of covariance showed that scores of the two groups for the PWBS scale and subscales did not reach a significant level, which indicates that there was no significant difference between the two groups in the posttest scores for the PWBS scale and subscales.

#### Life adjustment

The test of homogeneity of within-group regression coefficient reported that the scores of the experimental and control groups in the posttest on the SLAS scale and subscales, (F [1, 12] = 1.49, 0.026, 1.30, 1.92, 3.25, *p* > .05), did not reach a significant level, indicating that the slopes in the posttest of the experimental and control groups for the SLAS scale and subscales were the same. The result was consistent with the assumption of homogeneity of regression coefficient, and then we preceded to conduct analysis of covariance to compare the difference between the experimental and control groups in the pretest and posttest on the SLAS scale and subscales. However, the analysis of covariance showed that posttest scores of the two groups for the SLAS scale and subscales did not reach a significant level, which indicates that there was no significant difference between the two groups in the posttest scores for the SLAS scale and subscales.

### Analyses of the follow-up effects of the JBCP on anxiety, well-being, and life adjustment in Taiwanese children

#### Anxiety

The test of homogeneity of within-group regression coefficient reported that the scores of the experimental and control groups in the follow-up test on the SCAS scale and subscales, (F [1, 12] = 4.12, 0.002, 1.29, 0.04, 0.09, 1.16, 0.45, *p* > .05), did not reach a significant level. The result was consistent with the assumption of homogeneity of regression coefficient, and then we preceded to conduct analysis of covariance to compare the difference between the experimental and control groups in the pretest and follow-up test on the SCAS scale and subscales. However, the analysis of covariance showed that posttest scores of the two groups for the SCAS scale and subscales did not reach a significant level, which indicates that there was no significant difference between the two groups in the follow-up scores for the SLAS scale and subscales. As shown in Table 5, after eliminating the pretest effect, with the experimental treatment, the average scores of the experimental group on the follow-up test after adjusted for the SCAS scale and subscale were lower than those of the control group. However, one month after the experimental treatment, the overall anxiety level and other anxiety aspects in the experimental group participants were lower than the control group. This confirmed that the JBCP had a significant follow-up effect on anxiety in children.

**Table 5:** Means and Standard Deviations of the Adjusted Posttest of the Experimental Group and the Control Group for the Spence Children’s Anxiety Scale

Item	Experimental Group (*N* = 8)			Control Group (*N* = 8)		
	Pretest	Posttest	Posttest (Adjusted)	Pretest	Posttest	Posttest (Adjusted)
	M (SD)	M (SD)	M (SE)	M (SD)	M (SD)	M (SE)
Panic/ Agoraphobia	4.75 (4.27)	2.50 (2.93)	3.62 (1.40)	8.63 (4.27)	5.88 (5.46)	4.76 (1.40)
Separation Anxiety	6.25 (4.77)	5.50 (5.48)	5.55 (1.42)	6.38 (4.00)	5.75 (4.46)	5.71 (1.42)
Physical Injury Fears	4.63 (4.47)	3.50 (2.20)	4.26 (0.78)	8.13 (2.70)	5.75 (2.87)	4.99 (0.78)
Social Phobia	7.25 (4.20)	5.13 (3.68)	5.50 (1.32)	8.38 (5.29)	9.13 (5.69)	8.75 (1.32)
Obsessive Compulsive Disorder	5.25 (4.27)	2.86 (2.85)	3.50 (1.22)	8.38 (5.63)	5.50 (4.54)	4.88 (1.22)
Generalized Anxiety	4.25 (3.01)	3.63 (3.25)	4.61 (0.83)	6.25 (3.01)	6.88 (4.09)	5.89 (0.83)
Full Scale	32.38 (20.33)	23.13 (18.16)	28.61 (4.73)	46.13 (13.81)	38.86 (18.96)	33.39 (4.73)

#### Well-being

The test of homogeneity of within-group regression coefficient reported that the scores of the experimental and control groups in the follow-up test on the PWBS scale and subscales, (F [1, 12] = 0.006, 0.56, 1.54, 0.40, 0.20, 0.26, 0.18, *p* > .05), did not reach a significant level. The result was consistent with the assumption of homogeneity of regression coefficient, and then we preceded to conduct analysis of covariance to compare the difference between the experimental and control groups in the follow-up test on the PWBS scale. The analysis of covariance showed that follow-up scores of the two groups on the PWBS had no significant difference, but the control group significantly scored higher than the experimental group on the follow-test for the Purpose of Life subscale, (F [1, 13] = 8,96, *p* = .01, *p* < .05). The results showed that one month after the experimental treatment, the experimental group did not score better than the control group, while the control group scored better for the Purpose of Life than the experimental group.

#### Life adjustment

The test of homogeneity of within-group regression coefficient reported that the scores of the experimental and control groups in the follow-up test on the SLAS scale and subscales, (F [1, 12] = 0.79, 2.31, 0.22, 1.63, *p* > .05), did not reach a significant level. The result was consistent with the assumption of homogeneity of regression coefficient, and then we preceded to conduct analysis of covariance to compare the difference between the experimental and control groups in the pretest and follow-up test on the SLAS scale and subscales. The analysis of covariance showed that follow-up scores of the two groups on the SLAS and the subscales had no significant differences. The results showed that one month after the group counseling, the experimental group did not score better for school life adjustment.

### Qualitative data analysis

#### Group member feedback questionnaires

In the qualitative assessment of the group member feedback questionnaires, the average rating of the experimental group participants was above 3. Among them, 74.4% of the feedback were rated 4 (correct) and 5 (very correct). This demonstrates that the experimental group participants’ attitudes were very positive toward the JBCP sessions. The participants were very impressive with the program activities, such as Units “Knowing the Anxious Feelings,” “the Way of Relaxation,” “the Naughty Elf,” and “the Way the Brave talks.” Through learning, most participants felt that they knew themselves more during the JBCP sessions. They understood what initiated their cognitive thinking, emotions, and behaviors. They began to be more patient with some situations and knew how to cope with anxious feelings. Besides, they learnt how to express themselves. Overall, the experimental group participants found that the JBCP activities were helpful to them. As for the experience and feelings, the participants enjoyed the part where they played and interacted with other group members. They were impressed with the interactions with others. Therefore, most participants felt that they could sincerely express themselves. They trusted and cared about one another and felt freely and pleasantly to take part in the activities a group leader guided in the group. All above showed that the JBCP was beneficial to release anxiety in children and their self-acceptance, autonomy, and self-per relationship.

#### Semi-structured interviews with homeroom teacher

After the counseling treatment, we carried out content analysis to analyze semi-structured interviews. Five questions were addressed with analyses as follow:

(1) “Have the participants shared their experience and feeling of taking part in the group counseling sessions?” As for the participants experience sharing part: The participants were impressed with the interaction, conflicts, or some incidents happened during the Naughty Elf session. This indicated that the experimental group participants learned and gained something during the group activities. However, the participants’ emotions were also influenced by the group activities, conflicts, or incidents. Their opinions about the JBCP were that they enjoyed the group activities. However, they felt bothered for the group session tardiness of some participants or when their classwork or homework was not completed yet. This showed that the experimental group participants were motivated to take part in the JBCP activities and gained positive experience and feelings during the sessions. However, their performance may be interrupted by other participants or the need to adjust themselves to school or class regulations. (2) “Whether can the children clearly describe an incident’s cause and effect, their own thoughts, emotions, and reaction?” Apart from few participants with special needs and had difficulty expressing themselves or clearly describing an incident, most participants made progress in describing the cause and effect of an incident, expressing their own thoughts, emotions, or reaction. This indicates that the JBCP help to improve the cognition or expression ability of the experimental group members. After the participants reflect on their cognition, they became more aware of their own status within an incident and more comprehensively realizing themselves than before. (3) “After taking part in the JBCP sessions, do the children make any change in emotional expression?” Almost all the experimental group participants made some progress in their emotional expression, which shows that the JBCP successfully helped participants recognize, accept, and deal with their own negative emotions, such as anger, anxiety, etc. This also confirms that the JBCP has effects on anxiety and self-acceptance. Once the participants began to adjust and cope with their behavior to avoid conflicts with other people, they proved that the JBCP also has effect in autonomy and self-peer relationship. (4) “After taking part in the JBCP sessions, do the children make progress in study, interpersonal relationship, and school life? If they do, in what part have you ever noticed? What are the reasons you think may make them make progress? If they don’t, do you think what the reasons are?” As for interpersonal relation, most experimental group participants make different degree of progress, confirming the effect of JBCP on improving interpersonal relationship. As for study and life adjustment, even though the homeroom teachers did not see any change in academic performance or other life adjustment, still some participants improved their learning motivation. This shows that the JBCP might have effects on adjustment on academic performance, but there was no significant counseling effect. The homeroom teachers thought that three reasons motivated the participants. One was the participants’ caring and cooperation, another was the guidance and instructions given in the experimental group sessions, and the other was the time spent on practice and adjustment. The participants made progress under the cooperation among varieties of systems. They also implemented what they learned in the JBCP sessions into daily life and made some adjustments. Besides, the homeroom teachers stated two reasons why the experimental group participants failed to make progress in some parts. One was due to that some participants’ absence from and tardiness to school influenced their daily school assignment, they lacked the opportunities to learn and interact with peers. The other was due to that the participants were not ready and determined to make changes and take challenges. From this we can learn that the absence or tardiness influence the participants’ daily work, which may also influence the attendance to the JBCP. Besides, their mentally preparation also influenced their academic performance and interpersonal relationship within a group. (5) “Overall, what is the greatest benefit that the JBCP brings to the children?” The homeroom teachers referred to three beneficial parts. First, that the participants learnt better ways to adjust and express their emotions. Second, the participants learned to interaction with others and gained the opportunities to express their thoughts and feelings. Third, they were allowed to relax and take break and gained interpersonal and emotional support. This shows that the JBCP not only helps the participants learn how to express their emotions and interact with peers but also offer a secure and peaceful space for them to seek mental support from.

#### Group process note

We made a comprehensive analysis into the group progress notes taken during the 10 JBCP sessions:

#### Group participation

Some experimental group participants had difficulty attending all group sessions, which also made influence on other participants in their experience and learning. Besides, the group session arrangement was confined with school events. The sessions could not be scheduled in a continuous week for 10 weeks. Due to the long interval time span, it took time for the participants to warm up and get associated with previous JBCP sessions. The irregular JBCP session routine also influenced the session experience and effects of the JBCP.

#### Group participants’ performance

After each group activity, the counseling leader would rate the participants’ performance in the group (score 1 to 5 points). The participants’ performance including active participation, compliance with norms, careful sharing, active listening, and altruistic behavior. The experimental group participants’ performance in the group was above average. Most experimental group participants actively took part in group activities. They got high scores for active participation, following the rules, and being keen to share. The experimental group participants had stronger motivation. They followed directions and were willing to take part in group activities as well as actively express their thoughts. This demonstrates that the experimental group participants were carefree while being in the group and the atmosphere was active and cheerful. The experimental group participants scored lower for actively listening and altruistic behavior, which indicates that the participants were not good at waiting and listening to others. Some of them were eager to share their own thoughts and ignored other participants or ongoing activities, and this disturbance interfered with orders within the group and group progress. The low score for altruistic behavior shows that even though the experimental group participants positively interacted with others in the group and made positive feedback to others. They seldom reached out their hands to help other people, and some participants argued or had quarrels with other participants. These conducts greatly influenced the atmosphere in the group and group activities progress.

#### The reflection of group leaders

The JBCP guided the experimental group members to view events or emotions from a cognitive perspective and learn to understand the origin of the emotions to bring themselves new awareness and adjustment. However, the JBCP sessions also required the participants’ cognitive ability. Two units in the JBCP sessions, “Applied Skill-A Ladder for the Brave” and “an Ultimate Weapon of the Brave-How the Brave Thinks” needed one’s better cognitive abilities to understand. Therefore, it took more time to guide the participants to practice for a better perception and awareness. These two sessions demanded more counselors to guide the participants, but in the current study, there was only one group leader. Due to the limited attention, not all participants were always attended, lowering the benefits of the program to all participants.

## Discussion and implications

### Applicability of the SCAS in Taiwanese children

Study1 demonstrated that the SCAS scale and subscales corresponded with a medium to high reliability, which indicated that the SCAS had good reliability. Besides, the criterion-related validity analysis reported that the SCAS was highly correlated with the MASC-TV, which indicated that the SCAS had good validity. The reliability and validity tests both validated that the SCAS is a good instrument to assess anxiety in Taiwanese children.

The results of Study 1 reported that there was a significant difference in gender differences for the total scores of the SCAS. Girls had higher level of anxiety than boys in social phobia and generalized anxiety symptoms, indicating that girls have higher level of anxiety than boys. The results are similar to the anxiety-related studies in the past which mentioned that women tend to have a higher degree of anxiety than men. Women are likely to be diagnosed with anxiety, and this gender factor is also found in children.

As for ages, Study 1 explored the differences of similar ages and found that there was no significant difference between 11-year-old and 12-year-old children. However, except for the Obsessive Compulsive Disorder subscale, 12-year-olds averagely scored higher than the 11-year-olds in the Panic/Agoraphobia, Separation Anxiety, Physical Injury Fears, Social Phobia, and Generalized Anxiety subscales of the SCAS. The finding is similar to the study by Zhao et al. (2012) [53], in which they found adolescents had a higher degree of anxiety than children in China. Even though the education in Taiwan advocates adaptive education and diversified development to reduce the pressure on school children to learn and grow, the negative emotional pressures such as anxiety and tension of school children still increase with age, and it seems that the pressure of growth is still a big burden.

### The Effects of the JBCP on children’ anxiety

After the JBCP treatment, the posttest scores of the experimental group for the SCAS scale and subscales decreased. Also, with the pretest effect eliminated, the adjusted posttest means of the experimental group participants for the SCAS scale and subscales were lower than those of the control group children. The posttest score of the experimental group for the Separation Anxiety subscale was significantly lower than those of the control group. The group member feedback questionnaires and the participants’ homeroom teacher all confirmed that the anxiety feelings in the experimental group participants were relieved after receiving the JBCP treatment. This indicate that the JBCP had an immediate effect not only on children’s separation anxiety but also on their overall anxiety feelings. As for the follow-up test, the follow-up scores of the experimental group for the SCAS scale and subscales decreased. With the pretest effect eliminated, the adjusted follow-up mean of the experimental group for the SCAS scale and subscales were lower than shoes of the control group. This also confirmed that one month after the JBCP treatment had effects in overall anxiety feelings in children.

The current study showed that the JBCP specifically had immediate and significant effect on separation anxiety. We think this might be due to that separation anxiety symptoms are also found in children. March et al. (1997) [26] mentioned that children at the ages between eight to eleven have higher degree of separation anxiety disorder than children between 12 and 15 years old and between 16 and 19 years old, which indicated that children tend to have higher level of separation anxiety than children at other ages. Besides, the current study was conducted during the time when COVID-19 pandemic slowed down in Taiwan and many children began to go to physical class. Since children just returned to the campus and left their familiar home environment, they might have higher degree of separation anxiety. The JBCP treatment timely helped the children, so the JBCP had significant effects on separation anxiety symptoms.

In conclusion, the JBCP had immediate effects on lowering separation anxiety symptoms in children and had moderate effects on overall anxiety symptoms in children, confirming that the JBCP is a good counseling intervention tool.

### The Effects of the JBCP on children’ psychological well-being

After the JBCP treatment, there was no significant difference between the follow-up scores of the experimental group and the control group for the PWBS scale and subscales. However, the group member feedback questionnaires and the participants’ homeroom teachers all mentioned that the experimental group participants made progress in their self-acceptance, autonomy, and self-peer relationships. This indicates that the JBCP treatment has immediate effect on children’s well-being. There was no significant difference between the follow-up scores of the experimental group and control group for the PWBS scale and subscales, indicating that the JBCP has no follow-up effect on improving children’s psychological well-being.

We think that the current study aimed to explore psychological well-being, a realization of life meaning instead of temporary happiness, and it takes longer time and greater efforts to develop stable cognitive ability and emotions. Therefore, the immediate and follow-up effects of the JBCP were not significant. The well-being discussed in the current study is not a feeling of short-term happiness. It focused on the meaning of life. It would be better for the students to interact with others and rebuild the positive cognitive behavior pattern so that they can gradually internalize into a stable and positive behavioral pattern (Diener, 2000) [7].

In addition, the JBCP schedule arranged was confined with regular school routines and lasted only for 10 weeks. Compared with other counseling programs by Lin and Hsiu (2009) [25] and Lin (2011) [24], the time span of the JBCP is much shorter. Therefore, the time span spent on the JBCP also influenced the participants’ experience and learning. A limited time span and an unstable attendance rate of some experimental group participants both interfere with the experience of the participants who did not have enough practice for awareness and experience. Before the participants internalized what they learned and acquired during the JBCP sessions, the influence of the JBCP treatment on children’s inner psychological well-being is limited, and this influenced the consistent effects of the JBCP on the psychological well-being in children. With so many limitations, the JBCP developed in the current study still had immediate effects on children’s self-acceptance, autonomy, and self-peer relationship. Therefore, if children are granted more time to experience and practice, they may benefit more from the JBCP.

Besides, the control group scored significantly higher for the Life of Purpose subscale in the PWBS than the experimental group in the follow-up test. This indicates that one month after the control group participants received self-exploration and self-awareness practice, they internalized what they learned during the treatment sessions. They had greater and deeper understanding in the self-exploration and self-awareness which thus had follow-up effects on in children’s well-being. This also corresponds to the results of some relevant studies in the past on career and self-exploration (Güleç Keskin & Gülirmak, 2022; Sharma et al., 2023) [12] [33]. Genearlly speaking, the concept of positive psychotherapy could be useful to promote students’ development on spirituality level of subjective well being.

### The Effects of the JBCP on children’s school life adjustment

After the JBCP treatment, there was no significant difference between the posttest scores of the experimental group and the control group for the SLAS scale and subscales. The group feedback questionnaire and homeroom teachers mentioned that the experimental group participants developed a positive interpersonal relationship with their peers and their interpersonal interaction was also improved. This showed that the JBCP had effects on children’s school interpersonal relationship adjustment. As for the follow-up test, there was no significant difference between the follow-up scores of the experimental group and the control group for the SLAS scale and subscales, which indicates that the JBCP had no follow-up effect in children’s school life adjustment.

The reason why the JBCP did not have immediate and follow-up effects on the experimental group might be due to that a good school life adaption require consistent practice in school. The students need to constantly practice adjusting pressure to achieve a balance state between self and environment (Lazarus, 1976) [21]. Only by practice can children adjust well and persistently. However, due to the short time span of the program and the unstable attendance rate of some participants, the effects on children’s school life adjustment might be limited. Besides, some experimental group participants were students with special needs and some with tardiness problems. These issues may also cause challenges to adjustments on school life and academic performance. However, still some experimental group participants tried to apply the techniques they learned into life and improved their academic performance. This indicated that the JBCP still have effects on children’s life adjustment and learning.

### Limitations and suggestions

The limitations and suggestions are provided in the perspectives of counseling practice and research design. Counseling practice is related to experimental program and group leaders. The JBCP is an educational and counseling program that has preventive effects on emotional counseling. This program can be implemented in group counseling activities or in counseling and guidance class. However, we noticed that that JBCP required a certain degree of cognitive abilities in children. Therefore, we often needed more time to help the participants involved with deeper discussion and awareness. Besides, some incidents occurred during the JBCP sessions or conflicts between the participants shortened the time span of every session. With the limited time, we couldn’t carry out all group activities. Besides, the JBCP activities were confined with school events, which reduced the time for the experimental group participants to truly experience or practice all JBCP activities and influenced the effects of the JBCP. Therefore, we suggest a JBCP with a more extended time span for future, which allows program leaders to have more time to cope with incidents and arrange program focuses into different sessions. This may give group members have more time and opportunities to practice and adopt the experience they acquired into their daily life. Besides, a multi-assessment tool may understand group members learning experience and feels asl well as provide more opportunities for group member to implement into their lives.

As for group leaders for the JBCP, the leaders need to be competent in CBT knowledge and apply CBT skills to carry out the JBCP treatment, for the JBCP was designed and developed based on CBT. Therefore, we suggest the leaders to be prepared with CBT before the program begins. Besides, the JBCP sessions might need one more co-leader to conduct group activities. Because group leaders need to cope with different kinds of incidents during group sessions as well as attend to all group members needs. The group leader of the current study found that every participant had different cognitive abilities and ways of perceiving and responding to the surroundings. Some activities in the JBCP sessions, such self-exploration and thinking, required higher cognitive abilities to process. The JBCP participants needed guides and assistance to go through the activities, but the group leader of the current study found difficulty attending to all needs of the participants. In this case, future studies may include one co-leader to offer immediate assistance when needed. Besides, photos and records will be good materials to help the group leader to have a clear overview of overall and see the parts that can be easily missed.

Research design is divided into research tools, participants, methods. As for the research tools, we noticed that the instrument used in Study 2 consists of 93 items. The participants of the current study need to complete the instrument in the pretest, posttest, and follow-up test. The participants were found to lose their patience in the posttest and follow-up test and made complaints about the lengthy instrument. This might influence the results of the tests. Therefore, future researchers might need to be careful with tool selection and use the measure with less items to reduce the participants’ burdens. Besides, the participants were elementary school children in Taoyuan, Taiwan. Future studies may explore the effects of the JBCP on the participants at different ages and develop a counseling program for them. Regarding research methods, the current study aimed to explore the effects of the JBCP on anxiety, well-being, and life adjustment in Taiwanese children. Future studies may further conduct a case study to explore children’s internal change process to understand their change process and related impact factors.

## Data Availability

The datasets used can be accessed through the corresponding author.
